# Role of the Plasma Activation Degree on Densification of Organosilicon Films

**DOI:** 10.3390/ma13010025

**Published:** 2019-12-19

**Authors:** Rita C. C. Rangel, Nilson C. Cruz, Elidiane C. Rangel

**Affiliations:** Laboratory of Technological Plasmas (LaPTec), São Paulo State University (UNESP), Science and Technology Institute of Sorocaba (ICTS), Av. Três de Março, 511, 18087-180 Sorocaba, Brazil; rita.rangel@gmail.com (R.C.C.R.); nilson.cruz@unesp.br (N.C.C.)

**Keywords:** SiO_x_C_y_H_z_, HMDSO, PECVD, densification, organosilicon, corrosion barrier

## Abstract

The possibility of controlling the density of organosilicon films was investigated by tuning the plasma activation degree without providing extra energy to the structure, as usually reported in the literature. For this purpose, thin films were deposited in plasmas fed with hexamethyldisiloxane/Ar mixtures at a total pressure of 9.5 Pa. The power of the radiofrequency excitation signal, *P*, ranged from 50 to 300 W to alter the average energy of the plasma species while the electrical configuration was chosen to avoid direct ion bombardment of the growing films. In this way, it was possible to evaluate the effect of P on the film properties. Thickness and deposition rate were derived from profilometry data. X-ray energy dispersive and infrared spectroscopies were, respectively, applied to analyze the chemical composition and molecular structure of the layers. Surface topography and roughness were determined by atomic force microscopy while nanoindentation was used to evaluate the mechanical properties of the films. From electrochemical impedance spectroscopy the total resistance to the flow of electrolyte species was derived. The main alteration observed in the structure with changing P is related to the proportion of the methyl functional which remains connected to the Si backbone. Chain crosslinking and film density are affected by this structural modification induced by homogeneous and heterogeneous plasma reactions. The density increase resulted in a film with hardness comparable to that of the silica and more resistant to the permeation of oxidative species, but preserving the organosilicon nature of the structure.

## 1. Introduction

Organosilicons are amorphous materials, usually referred to as SiO_x_C_y_H_z_, considered as a specific class of (inorganic) polymers. This designation derives from their structures composed of a polysiloxane (–Si–O–)_n_ inorganic backbone surrounded by methyl functionals, forming methylsilyl (Si(CH_3_)_x_) groups. Weaker intermolecular forces differentiate them from other covalently bonded polymers, resulting in a structure with larger flexibility. In addition, methyl groups are free to rotate around the main backbone creating unoccupied volumes in the network, which have relevant implications for the material properties.

Thin films produced from organosilicons are normally characterized by high electrical resistance and optical transparency, in the visible region of the electromagnetic spectrum, enabling them for applications in microelectronics and integrated optics [1,2]. Furthermore, the combination of transparency, flexibility and moderate permeability to gas/vapor of such films favors a number of applications including encapsulation of organic electronic devices [3,4], improvement of packages performance (food, electrical devices, drugs) [5], reduction of water absorption and degradation [6,7], and protection of metal surfaces against corrosion [8,9]. The rotation of methyl groups may result in low surface tension materials, which are suitable for coating of fabrics [10,11], dental devices [12] and biocompatible [13] and anti-bio fouling surfaces [1,14].

Amongst the broad range of methodologies [1,15,16,17] used to grow SiO_x_C_y_H_z_ films, plasma-based deposition is very attractive for allowing the deposition of films with tailored properties by controlling the energetic conditions of the plasma and the reactions at the film-substrate interface [18,19,20,21]. In this context, several reports have demonstrated the suitability of plasmas containing hexamethyldisiloxane, HMDSO, ([CH_3_]_3_SiOSi[CH_3_]_3_), an inexpensive, easy to handle and nontoxic compound, to prepare transparent Si-based films. By varying the plasma conditions, which include deposition time, pressure and chemical composition of the gas mixture and power of the excitation signal, it is possible to deposit films with structures varying from predominantly (inorganic) oxide, SiO_2_ [22,23,24], to organosilicon [23,24,25,26], being the inorganic form denser, harder, scratch resistant and with better barrier properties. However, factors such as the adhesion of the silica to metal or polymer substrates, internal stress and especially defects generated in the deposition process or by substrate finishing affect the efficiency and physical stability of the oxide layer and, consequently, its practical application.

As stability problems are less common in organosilicon coatings [22,23,24,25,26], the improvement of its properties, mainly by the enhancement of density, becomes an issue of practical relevance. The densification of organosilicon films can be achieved by post-deposition treatments as reported, for instance, by Supiot et al. [27]. They have shown that organic groups are removed from organosilicon films exposed to oxygen plasmas, allowing the crosslinking of the structure. However, most of the proposed treatments caused the almost complete transformation of the organosilicon into an SiO_2_-like structure, which resulted on the same stability problems mentioned above.

The densification of organosilicon films deposited in remote thermal plasma was influenced by the substrate temperature. A reduction of the deposition rate with increasing process temperature is reported [28]. Under low deposition rates and higher temperatures, the residence time of the particles adsorbed on the surface increases, allowing the filling of the structure valleys. The result is the creation of denser films with fewer voids. In addition, ionic bombardment during film growth is also an important factor that provides extra energy and may lead to dissociation of agglomerates, contributing to structural densification [29]. In the work of Massier et al. [30] it is demonstrated that the temperature required for obtaining a film with high packing density can be reduced by associating ion bombardment to the deposition process. The enhancement of density of SiO_x_C_y_H_z_ films has also been proposed in the work of Blanchard et al. [21]. By changing the plasma excitation power and, consequently, the self-bias of the sample holder, the role of the power on plasma homogeneous (gas phase energetics) and heterogeneous (surface ion bombardment) reactions is discussed. The authors have proposed the densification is not entirely related to ion bombardment during film deposition but also to the plasma activation state.

Considering that the role of the plasma and plasma-surface reactions on the structure densification is not clearly understood and is still a subject under discussion [21,31], the main objective of this work is to investigate the densification of SiO_x_C_y_H_z_ films by changing the degree of plasma activation without providing extra energy to the structure, as normally reported in the literature. In this sense, an electrical configuration is proposed in which radiofrequency power is used to excite the plasma without enhancing direct ion bombardment of the growing layer. The excitation power has been chosen as the parameter to be varied since it impacts directly the energy of plasma species, and thus, may represent an easier way to densify the structure as compared to other procedures commonly used. Therefore, the specific hypothesis to be tested here is whether it is possible to control the crosslinking degree of organosilicon layers by adjusting the plasma power without inducing, deliberately, ion bombardment or heating of the substrates.

## 2. Materials and Methods

### 2.1. Experimental Apparatus and Procedures

**Figure 1:**
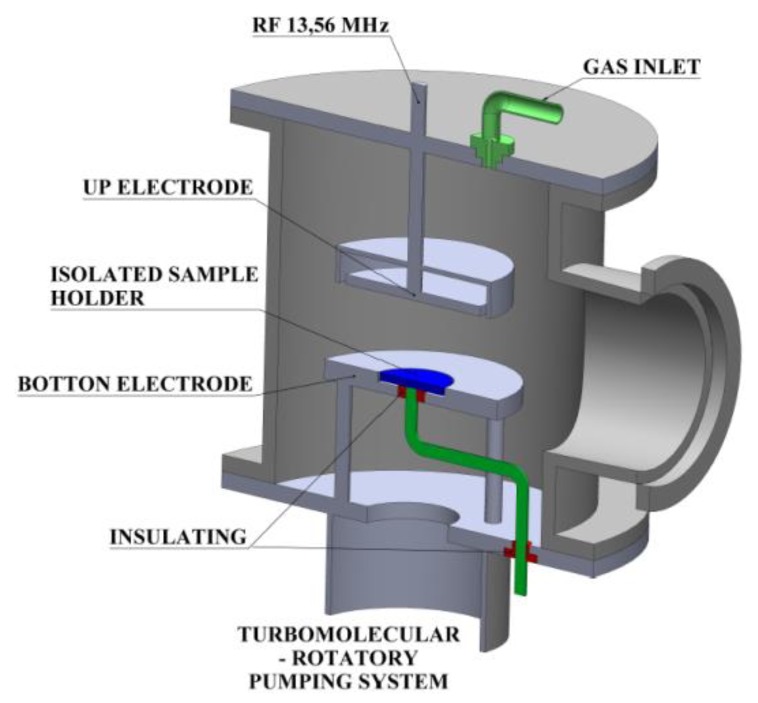
Experimental apparatus employed for film deposition.

Figure 1 shows the homemade system used for film deposition. It consists of a 6 L-stainless steel vacuum chamber fitted with two parallel circular electrodes, also of stainless steel. The bottom electrode is composed of two concentric discs of 16 cm (external) and 5 cm (internal) in diameter. The inner electrode, which is electrically isolated from external electrode, is used as sample holder.

Polished silicon and carbon steel specimens (99.630% Fe; 0.040% C; 0.008% Si; 0.240% Mn; 0.012% P; 0.006% S; 0.043% Al; 0.001% Cu; 0.005% Ni; 0.015% Cr) were the substrate materials. Before the experiments, all substrates were cleaned by sonication in ethanol and subsequently dried with N_2_.

The clean substrates were placed on the sample holder and system pressure was reduced down to 1.9 Pa with a rotary vane vacuum pump. The deposition atmosphere was created by introducing HMDSO (4 sccm) and Ar (45 sccm), producing a final pressure of 15.7 Pa with the aid of a throttle valve. The plasma was generated by applying a radiofrequency (RF) signal (13.56 MHz) to the upper electrode (10.5 cm in diameter), while the walls of the reactor and the external bottom electrode were grounded. Samples were maintained at floating potential during depositions. Deposition time was 900 s in all the experiments and the exciting power (*P*) ranged from 50 to 300 W.

### 2.2. Characterization Techniques

#### 2.2.1. Profilometer

Film thickness (*h*) was determined from the height of a step built by masking part of Si substrates with a glass slide during the depositions. The step height was measured in, at least, seven different regions with the aid of an Alpha-Step 500 KLA Tencor profilometer (KLA, Milpitas, CA, USA). Deposition rate (*R*) was evaluated as the ratio between thickness and deposition time.

#### 2.2.2. Infrared and X-ray Energy Dispersive Spectroscopies

The chemical structure of films deposited on polished silicon plates was analyzed by infrared reflectance-absorbance spectroscopy (IRRAS) in a spectrometer Bruker Vertex 70v (Bruker, Billerica, MA, USA). Spectra were recorded with resolution of 4 cm^−1^ in the range from 4000 to 375 cm^−1^. Each presented spectrum is the average of 32 scans acquired with each sample. The chemical composition was determined by X-ray Energy Dispersive Spectroscopy (EDS) using an X-ray Dry SD Hyper (EX-94410T1L11, JEOL’s DrySDTM, Peabody, MA, USA detector coupled to a scanning electron microscope JEOL JSM 6010 LA (Tokyo, Japan) using 4 keV electron beams.

#### 2.2.3. Contact Angle

Surface wettability was evaluated by contact angle measurements using deionized water as probe liquid. The presented results are the average (and standard deviation) of seven measurements in each sample using a goniometer Ramé-Hart 100-00 (Ramè-hart instrument co., Succasunna, NJ, USA) under controlled temperature and humidity conditions.

#### 2.2.4. Nanoindentation and Atomic Force Microscopy

Mechanical properties of films deposited on polished Si were measured by nanoindentation in a Hysitron TI 750 Ubi^TM^ system (Bruker, Minneapolis, MN, USA) fitted with a Performech^TM^ control scanning unit (Bruker, Minneapolis, MN, USA). Experiments were performed in 15 representative positions of each sample using a Berkovich tip and a maximum load of 5000 µN. Hardness and elastic modulus have been calculated from the unload portion of load-displacement curves using the model proposed by Oliver and Pharr theory [32]. Surface topography was inspected by atomic force microscopy (Bruker’s Hysitron TriboScope, Bruker, Minneapolis, MN, USA) using the nanoindenter and the same tip employed in the nanoindentation experiments. Scans were carried out over areas of 5 µm × 5 µm with a 2 µN normal force. The average quadratic roughness (RMS) was calculated from line profiles from three representative positions of each sample.

#### 2.2.5. Electrochemical Impedance Spectroscopy

Corrosion experiments were performed by electrochemical impedance spectroscopy (EIS) using a PARSTAT 4000 (Ametek Scientific Instruments, Oak Ridge, TN, USA) potentiostat/galvanostat with a three electrodes electrochemical cell, a frequency response analyzer and VersaStudio software (2.41.2). Samples were immersed in 3.5%, 0.6 M NaCl solution. Platinum/rhodium and silver plates were used, respectively, as counter and reference electrodes whereas the working electrodes were carbon steel substrates coated with the films deposited in this work. Data were acquired at open circuit potential, varying the frequency of the sinusoidal signal (10 mV) from 10^4^ to 10^−2^ Hz.

## 3. Results and Discussion

### 3.1. Thickness and Deposition Rate

**Figure 2:**
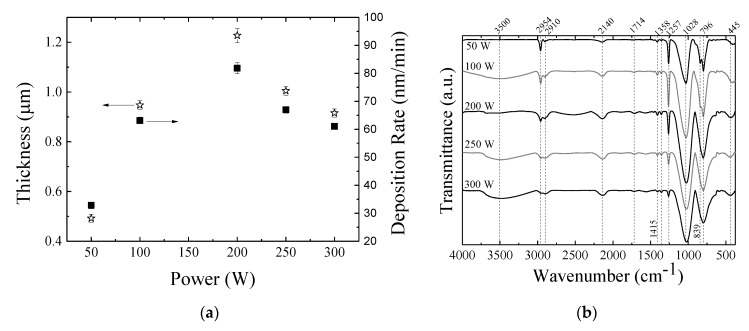
(**a**) Thickness and deposition rate of the films as a function of the plasma excitation power. (**b**) Infrared spectra of films deposited in plasmas excited with diverse powers.

Figure 2a shows thickness and deposition rate of the films as a function of the plasma excitation power. Since deposition time was kept constant (900 s) in all the experiments, *h* and *R* presented the same tendencies.

Both deposition rate and thickness increase with increasing power up to 200 W and then decreasing with the application of higher P. It is interesting to point out that, whenever the applied power was higher than 100 W, the deposition rate exceeded 60 nm/min. As a comparison, Vendemiatti et al. [6] reported a deposition rate of 21 nm/min for films deposited from atmospheres of 70% HMDSO, 15% O_2_ and 15% Ar applying 150 W of radiofrequency to the substrate holder. Similarly, Wavhal et al. [33] reported an increment in deposition rate, which ranged from 25 to 300 nm/min, of organosilicon films in plasmas from different mixtures of HMDSO and O_2_ as the power applied to the sample holder was increased. Despite the differences in electrical configuration and plasma composition, deposition rates obtained in the present work are within the range of values previously reported.

**Figure 3:**
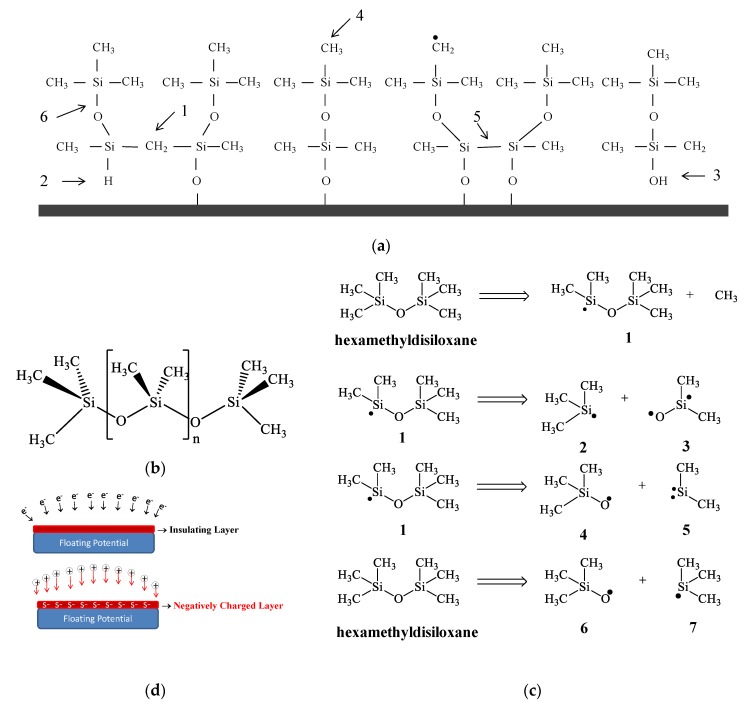
(**a**) Schematic representation of the structure of the films showing (1) chain crosslinking and the incorporation of (2) Si–H, (3) O–H and (4) C–H groups in an organosilicon structure. Adapted from Blanchard et al. [21]. (**b**) Chemical structure of the conventional polydimethylsiloxane, PDMS. (**c**) Chemical structure of hexamethyldisiloxane (HMDSO) and its possible fragmentation routes under the action of the plasma. (**d**) Charge annihilation mechanism in a dielectric surface induced by collisions of electron and subsequently of ions, as proposed by Yasuda [36].

The trend reversal observed in the curves of Figure 2a can be explained in terms of the dependency of the plasma parameters on *P*. The density of electrons with enough energy to cause activation of species increases with *P* when other plasma parameters are kept constant [34]. Thus, the overall plasma activity [35] is expected to increase with the increase of power intensifying all the processes, including the degree of monomer fragmentation. Under severe fragmentation, the deposition rate is expected to decrease [33] mostly due to gas evolution, resulting from the breakage of chemical bonds, and the consequent reduction of the size of molecular fragments available to be deposited on the substrate. Furthermore, as the density of charged species in the plasma is increased, another relevant factor, the ion bombardment, turns out to influence the deposition process. That is because, even in a floating potential, the impact of electrons on the insulating layer, creates a negative potential, the so-called self-bias potential, which accelerates the positive ions towards the sample holder [36] as schematically illustrated in Figure 3d. This mechanism contributes to charges annihilation, energy transfer and surface heating. Thus, film deposition takes place under ion bombardment. However, in such a case, the bombardment is ruled by the plasma activation degree as it depends on the negative potential developed on the insulating layer, which in turn, is a function of the density of plasma species. Therefore, it is expected a general increase of the bombardment intensity with increasing plasma activation and hence an increase of the sample holder temperature.

In polymeric structures, the deposition of energy by ionic collisions can induce ionization, atomic displacement, bond fragmentation and heating. The emission of chain termination and lateral groups, for instance, is a process that affects deposition rate not only because it causes species loss, but also chain crosslinking. Thus, the fall trends, observed in the curves of Figure 2a for applied power values higher than 200 W, can also be ascribed to a balance between homogeneous and heterogeneous plasma reactions and indicate a possible densification of the structure induced by crosslinking of chains.

### 3.2. Molecular Structure and Chemical Composition

Figure 2b shows the IRRAS spectra of the films. The wavenumbers of the main contributions and their correspondent assignments are summarized in Table 1. The emergence of the band at 3500 cm^−1^ indicates O–H bonds connected to Si [37]. The presence of Si–OH groups has important implications for the structure configuration and stability. Firstly, if OH binds to Si radical during the deposition (homogeneous reactions), it inhibits the establishment of connections between neighboring chains, resulting in a material with higher degree of porosity. Furthermore, Si–OH terminations represent a reactive point where degradation may initiate under the presence of water or moisture [38]. Both are detrimental points when barrier properties and chemical stability of the material are considered.

The presence of organic groups is indicated by the absorption bands at 2954, 2910, 1415 and 1358 cm^−1^, associated to stretching of C–H bonds in CH_3_ or CH_2_ groups. Interestingly, the intensity of these bands decreases even with the increase of film thickness as the power ranged from 50 to 200 W, pointing to a reduction in the concentration of organics with increasing *P*. All spectra revealed a contribution at 2140 cm^−1^ that is ascribed to Si–H stretching. Since this functional is not present in the precursor molecule, its presence indicates multiple-step reactions in the plasma phase.

Nevertheless, the main bands related to Si containing functionals appear in the low wavenumber region of the spectra. The band at 1028 cm^−1^, attributed to vibration of Si–O in Si–O–Si groups, is characteristic of HMDSO. A slight shift of this band to lower wavenumbers is observed with increasing *P*, indicating structure densification [39,40,41]. Since the shift is observed only for *P* > 200 W it correlates well with the reversal of the trends of thickness and deposition rate (Figure 2a).

Another important band appears at 1257 cm^−1^. This contribution, related to methylsilyl groups, Si–(CH_3_)_x_, indicates the formation of a structure composed by a main chain of Si–O–Si surrounded by methyl groups, independently of the deposition condition employed here. The resulting structure, schematically represented in Figure 3a, is very similar to that of conventional polydimethylsiloxane (Figure 3b). Furthermore, the comparison of the methylsilyl bands (1257 cm^−1^) in the spectra of the films prepared with 100 and 250 W, which presented roughly the same thickness, demonstrates an intensity reduction corroborating the idea of organics removal with increasing *P*.

The loss of methyl groups may arise from the homogenous plasma reactions by direct fragmentation of HMDSO as illustrated in Figure 3c. Under the impact of electrons [42], there is creation of a diversity of reactive fragments which can further react in the plasma phase generating groups with molecular weight higher than that of HMDSO [43].

The fragments can also connect directly to the surface acting as a precursor of film growth. The resulting structure will depend on the fragmentation-recombination degree of the species in the plasma phase (homogenous reactions) as well as the plasma-surface reactions (heterogeneous reactions). It means that ion bombardment induced by charges accumulation can also remove lateral groups and chain terminations such as H or even CH_3_. According to Rao [44], the reduction in the intensity of the methylsilyl band is also associated with the increase of the structure crosslinking. Thus, it is postulated here an increment in the density of the films with increasing *P* due to an enhancement of the crosslinking degree. This result is in accordance with the shift of the band at 1028 cm^−1^ as mentioned above and explains, together with the loss of molecular –(CH_3_)_x_ fragments, the fall in the deposition rate and in the film thickness for the depositions with applied power higher than 200 W.

Groups Si(CH_3_)_x_ are also identified by the bands at 839 (Si(CH_3_)_3_) and 796 (Si(CH_3_)_2_) cm^−1^. The enlargement and loss of resolution of these bands suggest changes (disordering) in the vicinity of the chemical bonds due to greater diversity of fragments incorporated in the structure [37]. The band at 796 cm^−1^, which may also have the contribution of stretching vibration of Si–O in Si–O–Si [45], indicates the presence of the Si(CH_3_)_2_ chain propagation unit, characteristic of the PDMS structure. High proportions of this group suggest formation of a structure with long chains. As illustrated in the schematic representation of the structure in Figure 3a, chain interconnection may occur via Si–CH_2_–Si (indicated by 1 in Figure 3a), Si–Si (indicated by 5) and Si–O (indicated by 6 in Figure 3a) groups. It shows that, the loss of H itself may favor the connection of neighboring chains. Nevertheless, all of the illustrated possibilities involve a reduction in the proportion of methyl groups of the film. The absorption of O–H group by pendant bonds (point 3 in Figure 3a) thus inhibits chain reticulation.

**Figure 4:**
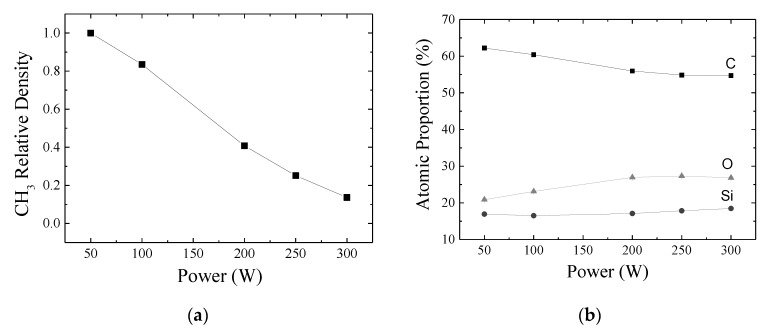
(**a**) Relative density of Si(CH_3_)_x_ groups in the films as a function of *P*. Data obtained using the model proposed by Lanford and Rand [46] and the intensity of the absorption band at 1257 cm^−1^ in Figure 2b. (**b**) Atomic proportion of C, O and Si determined by Energy Dispersive Spectroscopy (EDS) in the films as a function of *P*.

Figure 4a shows the relative density of Si(CH_3_)_x_ groups as a function of *P*, calculated by the method proposed by Lanford and Rand [46] considering the absorption band at 1257 cm^−1^. The relative density of Si(CH_3_)_x_ groups progressively fall with *P*, confirming the loss of CH_x_ and H groups. Chain crosslinking becomes probable, depending on the concentration and proximity of the radicals. When dangling bonds are not consumed by crosslinking, they remain active and react with atmospheric groups when the sample is exposed to air. This mechanism partially explains the presence of Si–OH functional in the organosilicon structure. The residual atmosphere in the deposition chamber (1.9 Pa) and the presence of O in the precursor compound may also account for O–H incorporation during the film deposition (homogeneous reactions).

Figure 4b shows the proportions of C, O and Si in the films as a function of *P*, derived from EDS data. Together with the information on chemical structure, the detection of these species corroborates the proposal of an organosilicon material. Proportions very similar to these, derived from XPS (X-ray photoelectron spectroscopy) results, were reported for organosilicon films in the work of Blanchard et al. [21] and Montarsolo et al. [47]. Furthermore, considering the chemical structure of the conventional PDMS, which is schematically depicted in Figure 3b, and disregarding H that is not detected by EDS, structures containing 50% C, 25% O and 25% Si are expected while the HMDSO molecule, which is depicted in Figure 3c, contains 67% C, 11% O and 22% Si. Therefore, the precursor molecule should undertake reactions for carbon abstraction, resulting in the film stoichiometry observed here. The small fragments generated by homogeneous or heterogeneous reactions may undertake multiple step reactions in the plasma, resulting in stable neutral species, such as, C_2_H_2_, CH_4_, C_2_H_6_ and C_3_H_8_, or may be further oxidized by residual atmosphere in the reactor, generating, for instance, CO, CO_2_, OH or H_2_O groups. All of them are very prone to be removed from the chamber by the vacuum system.

As a necessary condition for developing the study proposed here, the organosilicon nature of the material is preserved with the enhancement of *P*. Only slight variations are detected indicating reduction in the proportion of C and elevation in the contents of Si and O. Interestingly, the variations of the proportions of C and O have roughly the same intensity. However, the contrary trends show that the variations are caused by the same phenomenon. In other words, the loss of methyl groups decreases the proportion of C in the film, which, in turn, causes the O content to proportionally increase. Therefore, it can be inferred from results of Figure 4b that it is possible to slightly change the chemical composition of organosilicon films by controlling the plasma activation degree. It is further observed that the system is operating in a low energy regime once the increment in the energy supplied to the plasma enhances the deposition rate (in the range 50–200 W), while preserving the same nature of the precursor fragments.

Therefore, the overall analysis of the molecular structure and chemical composition revealed a material composed by C, H, O and Si organized as an organosilicon structure. Structural densification induced by crosslinking is suggested by the shift of the Si–O band (1028 cm^−1^) towards smaller wavenumbers, by a reduction of the intensity of the bands related to the methylsilyl functionals and of the proportion of C with the elevation of *P*. Alterations in the porosity, mechanical and barrier properties are expected as a consequence.

### 3.3. Wettability and Surface Topography

**Figure 5:**
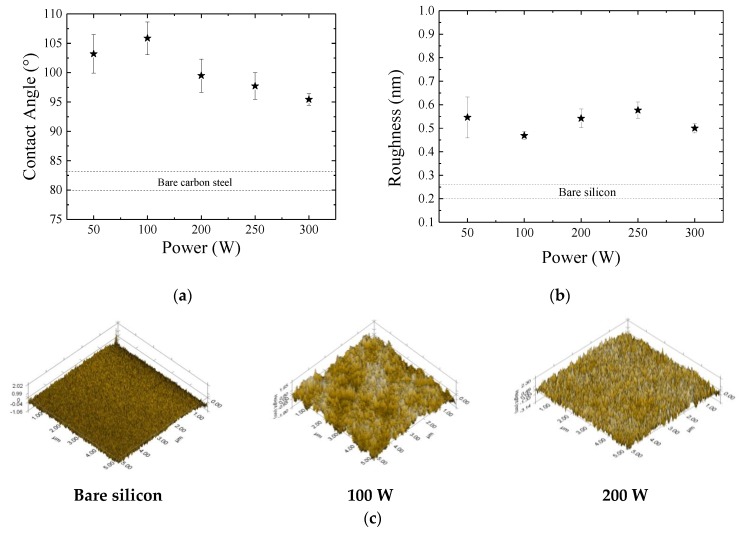
(**a**) Contact angle of the films as a function of the plasma excitation power. (**b**) Average quadratic roughness (RMS) of the films as a function of *P*. Dotted lines represent the contact angle and roughness for the untreated substrates. (**c**) Atomic force microscopy three-dimensional (3D) topographic profiles (5 μm × 5 μm) of the samples prepared in plasmas with 100 and 200 W, as well as of the bare silicon substrate.

Figure 5a shows the contact angle, *θ*, of the films as a function of *P*. The dotted lines present the results for the bare carbon steel. After film deposition all the surfaces became hydrophobic with contact angles ranging from 90 to 110°. The comparison of such results with those obtained for polytetrafluorethylene, Teflon^®^, (*θ* ~100–115°) [48] indicates the formation of non-polar surfaces upon film deposition. In general, films deposited using the lowest powers (50–100 W) are less receptive to water than those deposited using higher *P*.

Results in the literature show significant differences between the contact angle of silicon oxide and organosilicon films [6,14,49]. The former is hydrophilic due to high concentration of Si–O, favoring the incorporation of OH groups. The uptake of water molecules by hydroxyls is then induced by Keesom interactions. Although organosilicon films also present Si–O functionals, these bonds are shielded by nonpolar methyl groups (–CH_3_), which rotate to the air-film interface, causing a reduction in the electrostatic forces acting on the water molecules [50]. Thus, the higher contact angle of the samples prepared with the lowest powers (50 and 100 W) is consistent with the preservation of the CH_3_ groups on these surfaces, in accordance with the structure proposed in Figure 3a. On the other hand, the decreasing in *θ* for *P* > 100 W confirms the abstraction of such shielding groups, which affects the surface polarity.

Since roughness can affect surface wettability, the root mean square roughness (RMS) of the films was determined and is presented in Figure 5b as a function of *P*. Dotted lines in this graph represent the roughness values for the silicon substrate (0.20 to 0.23 nm). Surface roughness increases upon film deposition, attaining values (~0.55 nm) similar to those reported in the literature for organosilicon films with the same thickness [22,51]. It is readily observed in this graph that power variation does not affect significantly the roughness of the films.

These results are understood taking into account the three-dimensional (3D) topographic profiles of the samples, depicted in Figure 5c. While the bare Si plate is smooth, the surfaces of coated samples are no longer flat due to corrugations created by peaks and valleys. The asperities, formed by agglomerates with hundreds of nanometers in size, have already been detected in previous works [22,51]. According to Huan et al. [51], the geometry and concentration of post-like structures straightly affect the coating porosity. Such structures are formed by oligomerization of the HMDSO fragments in the plasma, generating high molecular weight-macromolecules that reach the substrate and stay connected to it by van der Waal’s or chemical bonds. Moreover, voids, pinholes, radicals and micro-cracks [52,53], induced on the surface by ion bombardment, may act as preferential sites for the film nucleation explaining the rather uniform aspect of the surfaces with increasing bombardment intensity. Chain crosslinking, however, is pointed as the main responsible mechanism for the surface smoothening. However, despite the agglomerates, the films cover uniformly the entire inspected areas with no signal of detachments, cracks or uncoated regions. Thus, the clear transition from rougher to smoother surface, demonstrated by disappearance of the cavities (darker regions in Figure 5c) with the elevation of the power beyond 100 W, is further evidence of the structural crosslinking induced by changes in *P*. Finally, no clear influence of the roughness on the contact angle is observed with the variation of *P*. Therefore, chemical composition is pointed as the main responsible for the change of the wettability.

### 3.4. Mechanical and Barrier Properties

**Figure 6:**
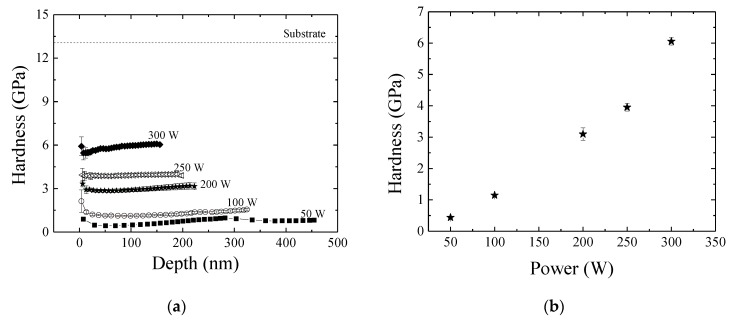

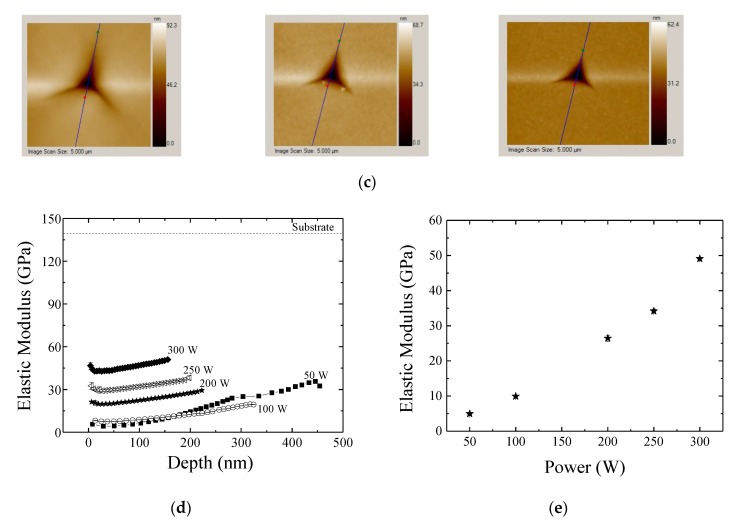
(**a**) Hardness as a function of depth for films deposited in plasmas of different powers. Dashed line corresponds to the average hardness for the bare silicon substrate. (**b**) Hardness of the films as a function of the plasma excitation power. (**c**) Topographic profiles in 5 µm X 5 µm area of the samples prepared in plasmas of 50, 200 and 300 W, from left to right, respectively. (**d**) Modulus of elasticity as a function of depth for films deposited in plasmas of different powers. Dashed line corresponds to the average elastic modulus for the bare silicon substrate. (**e**) Elastic modulus of the films as a function of plasma excitation power.

Figure 6a shows the hardness of the films as a function of depth (the distance from the film surface). Dashed line represents the average hardness for the silicon substrate, which is in good agreement with literature [54]. Surfaces softer than Si were produced in all cases, but a straight dependency of hardness with the plasma power can be observed as the curves shifted upwards with increasing *P*. Furthermore, a slight increasing in hardness is observed in all the individual curves with increasing depth, revealing the interference of the mechanical properties of the substrate on the results. To minimize such interference, hardness was determined at depths corresponding to 15% of the films thickness [55,56,57]. Results are shown in Figure 6b as a function of *P*.

Soft films (0.4 GPa) were deposited under the lowest power condition confirming the organosilicon nature of the structure [58,59]. However, a continuous elevation is observed in the hardness curve with increasing *P*. For films deposited under the highest power condition, hardness reached 6.0 GPa, which is 93% higher than that of the softest film. As a reference, plain carbon steels have hardness ranging from 2.0 to 3.0 GPa [60] while the hardness of tool steels ranges from 5.0 to 12.0 GPa [57,61]. The increasing of hardness with *P* is confirmed as one analyze the dimensions of the indentations produced using the maximum indentation load (5000 µN) on the different surfaces as shown in Figure 6c, where a reduction in the strain area can be clearly observed, with increasing *P*.

Even though the organosilicon nature of the films is not altered by varying the plasma activation degree, mechanical resistance is significantly improved. This result corroborates the idea of increment in the reticulation degree and evidences the importance of this characteristic for the structure properties. Aside to decreasing the distance of neighboring chains, crosslinking generates anchoring points, which increase the connectivity of the inorganic backbone, turning the material more resistant to mechanical requirement. The anchoring points also tend to reduce the flexibility of elastomeric systems, such as rubbers or the silicone-like films deposited here under low activation powers, resulting in more rigid structures. To evaluate this aspect, the reduced elastic modulus of the films, *E_r_*, was evaluated and is presented in Figure 6d as a function of depth. The average *E_r_* for the Si substrate is represented by the dotted line. In general, curves shift upwards with increasing *P*, indicating the enhancement of the structure rigidity. However, the ascending trend of each individual curve with increasing depth indicates, by itself, interference of the mechanical properties of the substrate. In order to minimize such interference, *E_r_* values were determined at depths corresponding to 15% of the total film thickness (Figure 6e). The elastic modulus increases by almost an order of magnitude (from 5.0 to 49.0 GPa) with increasing *P* from 50 to 300 W. Reference for these values is provided in the work of Coclite et al. [62] which prepared SiOx films with *E_r_* = 90 GPa. That is, it is possible to prepare organosilicon structures with rigidity similar to that of silica by increasing the degree of crosslinking.

**Figure 7:**
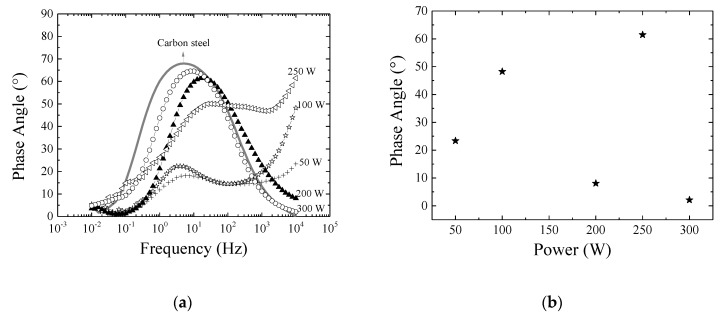

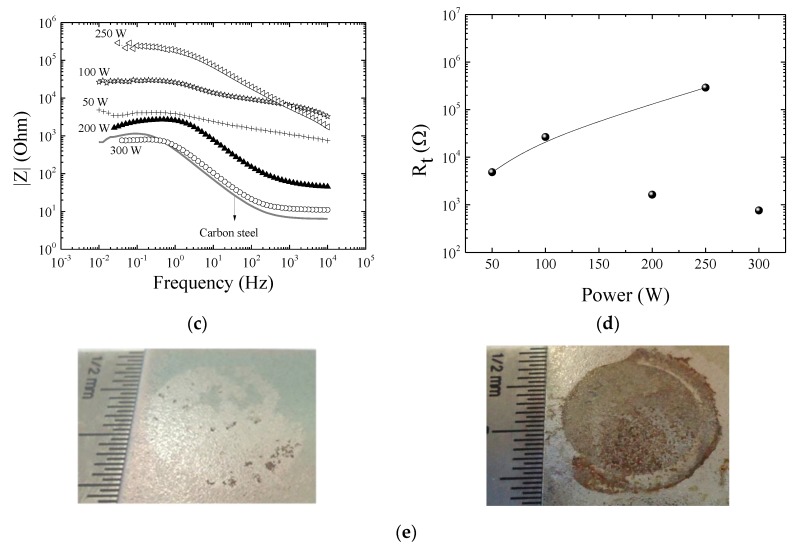
(**a**) Phase angle as a function of the frequency of carbon steel samples as-received and after plasma treatment with diverse applied power. (**b**) Phase angle of the films at 10^4^ Hz as a function of *P*. At this frequency, the phase angle for the bare carbon steel is 0°. (**c**) Impedance modulus as a function of the frequency for the bare and coated carbon steel substrates. (**d**) Total resistance of the system as a function of *P*. (**e**) From left to right, photographs of the tested regions on the samples prepared under 100 and 300 W of power, respectively.

Figure 7a shows the phase angle for bare and coated carbon steel samples, as a function of polarization frequency. The curve with one concavity is characteristic of the untreated substrate [63]. The results obtained with the samples produced under 200 and 300 W are very similar to those found for the bare carbon steel. On the other hand, two concavities and displacement of the high frequency tail to higher phase angles, both revealing the presence of the coating, can be observed on the curves of the other samples.

Capacitive layers are expected to better inhibit the transport of electrolyte species. Since in capacitive systems the phase angle between current and tension is 90°, samples with phase angles close to this value are expected to better prevent the flow of electrolyte species. The phase angle of the systems at the highest frequency edge (10^4^ Hz) is presented in Figure 7b as a function of *P*. A null value of phase angle was detected for the bare substrate while reduced values are observed for the samples prepared with *P* equal to 200 and 300 W. If these two samples are not taken into account, a continuous rising in phase angle is observed with increasing *P*. Visually inspecting the tested area of these two samples in the photographs of Figure 7e, it is clearly evidenced the detachment of the films during the electrochemical experiments. This finding indicates the formation of stressed structures for the thickest (200 W) and more crosslinked (300 W) films. Although the results obtained by nanoindentation revealed well adhered films in all the cases, for the corrosion analysis the films were deposited on carbon steel instead of silicon and immersed in saline water solution, factors that may influence the adherence. The adhesion of the layer to the substrates, internal stress and mainly defects generated during the deposition process affect the chemical stability and effectiveness of the protective layers [22,49]. The general increasing of the phase angle with *P* observed on the coatings which presented physical stability to the corrosion test, can be ascribed to a reduction of the concentration and/or mobility of the species going through the film, caused by the structural densification. Thus, phase angle results corroborate the assumption of structural densification induced by chain crosslinking.

Figure 7c shows the impedance modulus, |Z|, as a function of the frequency for the samples investigated here, including the bare carbon steel (solid line). The curves for the samples prepared with 200 and 300 W of power present just slight differences with respect to that of the carbon steel, consistently with the observed peeling off of these films. For the other curves, an upward shifting is observed with increasing *P*, showing an overall improvement of the corrosion resistance. To quantify this improvement, the total resistance [64], R_t_, of the systems was determined, as shown in Figure 7d as a function of *P*. If the results of the samples which peeled off are disregarded, an increase in R_t_ is obtained with increasing *P*, in a way very similar to that observed for the phase angle. Specifically, R_t_ is about 60 times higher in the system prepared in highly activated plasma (250 W) than in the less activated one (50 W), showing the importance of the densification induced by crosslinking for the current flow in the circuit.

Therefore, from the phase angle and impedance modulus results, it is possible to infer that permeation of species through the deposited layer is changed increasing the plasma power since it straightly affects the connectivity of the organosilicon network.

## 4. Conclusions

All the deposition conditions employed here resulted in surfaces uniformly coated independently of the substrate material. No signals of detachment, cracks or uncoated regions were observed in the inspected areas.

Under the conditions used here, the deposition rate and layer thickness are sensitive to the plasma power while molecular structure and chemical composition are just barely affected. In general, films are composed by C, H, O and Si, ordered as an organosilicon network. The loss of H and methyl is the main route of alteration, influencing the overall structure connectivity. The intensity of ion bombardment and the type of film precursor species rule such modifications. Therefore, changes in homogeneous and heterogeneous reactions of the plasma slightly alter the deposition kinetic but have important implications on the material properties.

The results of water contact angle measurements coordinate with the reduction in the concentration of non-polar groups (methylsilyl) responsible for the electrostatic shielding of the polar Si–O–Si backbone. Roughness changed after film deposition but without affecting the water contact angle, ruled majorly by chemical rather than physical aspects. Furthermore, the reduction in the coating porosity with the elevation of power was suggested by the surface smoothening.

The plasma power affects significantly the mechanical and barrier properties of the films. Harder and less flexible structures are created as connectivity increases. For treatments with excitation power higher than 200 W, the crosslinking increases substantially, making the film more stressed and, therefore, inappropriate for application as protective coatings.

The corrosion resistance of the carbon steel was improved by the film deposition in all the cases. However, with the improvement of the organosilicon crosslinking degree, the total resistance increased 60 times. All these modifications were implemented without changing the nature of the films, demonstrating the possibility of production of denser and harder organosilicon barrier layers with properties very similar to those of silica by tuning the plasma activation degree.

Finally, it should be stated that the films developed herein may be reproduced on other types of substrates (i.e., ceramics, glasses, polymers and other metallic surfaces) for different types of applications.

## Figures and Tables

**Table 1:** Wavenumber and assignments of the bands detected in the infrared absorption spectra of the films.

Wavenumber (cm^−1^)	Mode
3500	OH in Si–OH
2954	ν C–H in CH_3_ and CH_2_
2910	ν C–H in CH_2_
2140	Si–H
1714	ν C=O in CH_2_O
1415	δ C–H in Si–(CH_3_)_x_
1358	δ C–H_2_ in Si–CH_2_–Si
1257	δ C–H_3_ in Si–(CH_3_)_x_
1028	ν Si–O in Si–O–Si and Si–O–C
839	ν Si–C in Si–(CH_3_)_3_
796	ν Si–C in Si–(CH_3_)_2_ and Si–O in ν Si–O–Si
445	Si–O–Si (bending)
